# From Acute Symptomatic Seizures to Post-Stroke Epilepsy: A Narrative Review of Diagnosis and Management

**DOI:** 10.3390/jcm15145349

**Published:** 2026-07-08

**Authors:** Tomásia Frezatti, Leonardo Roever, Octávio Pontes-Neto

**Affiliations:** Department of Neuroscience and Behavioral Sciences, Ribeirão Preto School of Medicine, University of São Paulo, Ribeirao Preto 14040-900, SP, Brazil; leonardoroever@hotmail.com (L.R.); opontesneto@fmrp.usp.br (O.P.-N.)

**Keywords:** post-stroke epilepsy, acute symptomatic seizures, stroke, antiseizure medication, seizure prophylaxis, epileptogenesis, risk stratification, cerebrovascular disorders

## Abstract

Stroke is a major cause of acquired epilepsy in adults, particularly in older individuals. However, seizures after stroke should not be regarded as a single clinical entity. Acute symptomatic seizures (ASyS) occur within the first seven days after stroke and are considered provoked events, whereas remote symptomatic seizures carry a higher recurrence risk and may fulfill the practical definition of post-stroke epilepsy (PSE). This narrative review provides a clinically oriented synthesis of current evidence on definitions, epidemiology, pathophysiology, risk factors, predictive scores, EEG and biomarker-based risk stratification, seizure prevention, and antiseizure medication (ASM) management. Current evidence does not support routine primary antiseizure medication prophylaxis for all stroke patients. In contrast, documented clinical or electrographic seizures require appropriate treatment, and established PSE often requires long-term individualized therapy. Predictive tools may help guide surveillance, EEG indication, counseling, and follow-up, but should not be used as automatic triggers for prophylactic treatment. ASM choice should account for seizure type, age, comorbidities, cognitive and psychiatric vulnerability, drug interactions, and secondary vascular prevention. Future research should focus on validated biomarkers and preventive strategies capable of modifying epileptogenesis after stroke.

## 1. Introduction

Stroke is one of the leading causes of acquired epilepsy in adults and a major cause of new-onset epilepsy in older individuals, particularly after the age of 65 years [1,2,3]. However, seizures occurring after stroke should not be automatically interpreted as post-stroke epilepsy (PSE). Their classification depends primarily on the temporal relationship with the vascular event. Acute symptomatic seizures (ASyS) occur within the first seven days after stroke onset and are considered provoked events; therefore, they do not fulfill criteria for epilepsy. In contrast, remote symptomatic seizures occur later (>7 days) and carry a substantially higher risk of recurrence. In stroke survivors, a single remote symptomatic seizure, since unprovoked, fulfills the practical definition of epilepsy proposed by the International League Against Epilepsy (ILAE) and justifies consideration of long-term antiseizure medication (ASM) treatment [1,4,5]. These definitions directly influence prognosis, counseling, treatment duration, and follow-up strategy.

The frequency of ASyS and PSE varies according to stroke subtype, lesion characteristics, clinical severity, patient profile, and diagnostic strategy. Overall, seizures and epilepsy are more frequent after hemorrhagic than ischemic stroke [6,7]. In intracerebral hemorrhage (ICH), ASyS occurs in approximately 6–15% of patients, most often within the first 72 h [8]. In ischemic stroke, reported frequencies are generally lower, although estimates vary substantially across cohorts. Acute symptomatic status epilepticus is uncommon, affecting approximately 0.1–0.3% of patients with ischemic stroke, but is associated with particularly poor prognoses [1].

Part of this variability reflects differences in seizure ascertainment. Subtle focal seizures, nonconvulsive seizures, and electrographic-only seizures may be missed in routine clinical series, particularly when systematic electroencephalographic (EEG) monitoring is not performed. In a video-EEG study of patients with acute anterior circulation ischemic stroke monitored during the first 72 h, early seizures were detected in 14.6% of patients, including electrographic-only seizures in almost one quarter of cases [9]. These findings support active surveillance in selected high-risk patients but should not be directly extrapolated to unselected stroke populations. For established PSE, cohort studies report risks of approximately 3.0–8.7% after ischemic stroke and 7.6–15.4% after hemorrhagic stroke, with the highest risk concentrated within the first year and declining thereafter [3,10,11].

Although most available evidence derives from adult populations, post-stroke seizures are also clinically relevant in children. The risk of epilepsy is particularly increased after perinatal ischemic stroke and may remain elevated decades after the initial insult [12,13]. In neonates and children with cerebral venous thrombosis (CVT), ASyS occurred in approximately one quarter of patients in one cohort and was associated with subsequent epilepsy risk [14]. These observations reinforce that age, stroke mechanism, and vascular subtype should be considered when interpreting seizure risk after cerebrovascular injury.

Seizures after stroke are not only diagnostic events but also prognostic markers. They are associated with cognitive decline, dementia, poorer functional outcomes, increased mortality, greater resource utilization, and longer hospital stay [2,15,16,17]. In patients with PSE, seizure recurrence has been independently associated with functional decline, including deterioration in modified Rankin Scale scores [18]. In children, PSE may also be associated with poorer neurological outcomes, and patients with severe underlying conditions appear to be at increased risk of drug-resistant epilepsy [19,20].

Despite this clinical relevance, management of seizures after stroke remains challenging. Current evidence does not support routine primary ASM prophylaxis for all stroke patients, and available guidelines emphasize the limited quality of evidence in this field. Conversely, long-term treatment is usually considered in patients with established PSE [21]. Therefore, clinical decision-making requires more than recognition of seizures after stroke; it requires careful distinction between ASyS and established PSE.

This narrative review integrates current evidence on definitions, epidemiology, risk stratification, diagnostic evaluation, seizure prevention, prophylactic treatment, and ASM management. Its aim is to provide a clinically oriented framework for recognizing, predicting, and managing seizures across the continuum from acute symptomatic seizures after stroke to established PSE.

## 2. Methods

This narrative review was based on a structured literature search of PubMed/MEDLINE (National Library of Medicine, Bethesda, MD, USA) and Embase (Elsevier B.V., Amsterdam, The Netherlands). The research focused on articles in English published up to April 2026, including human studies, reviews, meta-analyses, and guideline documents. The main search terms were: “post-stroke epilepsy”, “post-stroke seizure”, “stroke and epilepsy”, “stroke and seizure”, and “cerebrovascular disease and epilepsy”. Search results included nearly 8000 manuscripts published in the past ten years. Animal studies were excluded. Because this was a narrative rather than a systematic review, formal duplicate removal and PRISMA-style record tracking were not performed. Reference lists of selected articles were manually screened to identify additional relevant publications.

Titles and abstracts were screened for relevance. Priority was given to articles addressing post-stroke seizures (PSE) in relation to definitions, epidemiology, risk stratification, prediction models, EEG and neuroimaging findings, prevention strategies, prognosis, and ASM management. Studies focused on stroke without specific discussion of seizures, epilepsy, epileptogenesis, diagnostic evaluation, or treatment implications were excluded unless directly relevant to the conceptual or therapeutic framework of the review. The final reference list was selected to support a clinically oriented synthesis of the continuum from acute symptomatic seizures to established PSE.

During the preparation of this manuscript, the authors used ChatGPT, GPT-5.5 Thinking (OpenAI OpCo, LLC, San Francisco, CA, USA) for English-language editing and AI-assisted tools to support the initial graphical drafting of figures. All AI-assisted outputs were manually reviewed, corrected, and finalized by the authors, who take full responsibility for the content of this publication.

## 3. Epilepsy and Stroke: A Bidirectional Relationship

The relationship between stroke and epilepsy is increasingly recognized as bidirectional. While stroke is a well-established cause of acquired epilepsy, accumulating evidence suggests that people with epilepsy also have an increased risk of cerebrovascular events. This association has been supported by observational cohorts and, more recently, by a Mendelian randomization study, a genetic epidemiology approach that uses genetic variants as instrumental variables to support causal inference between exposures and disease outcomes [22].

In several cohorts, people with epilepsy have shown an approximately two-fold increased risk of stroke compared with individuals without epilepsy [23]. This higher risk is likely multifactorial and may reflect shared vascular risk factors, systemic pro-inflammatory states, and the metabolic effects of some ASMs [22]. In particular, enzyme-inducing ASMs may adversely affect cardiovascular risk profiles by promoting dyslipidemia and increasing homocysteine and C-reactive protein levels, thereby potentially contributing to atherosclerosis [24].

This issue appears especially relevant in older adults with epilepsy, in whom baseline vascular risk is already high. In a cohort evaluating cardiovascular events, including stroke, transient ischemic attack, and myocardial infarction, older people with epilepsy had an approximately two-fold increased risk of cardiovascular events, and nearly one-third of this association was attributed to enzyme-inducing ASM exposure [24].

Therefore, the bidirectional relationship between epilepsy and stroke has practical implications: clinicians should not only recognize stroke as a major cause of late-onset epilepsy but also consider vascular risk assessment and ASM choice as part of the long-term care of people with epilepsy, particularly in older patients or those with additional cardiovascular risk factors.

## 4. Pathophysiology and PSE Biomarkers

The mechanisms underlying poststroke epileptogenesis are complex, multifactorial, and incompletely understood. From a clinical perspective, it is useful to distinguish mechanisms that favor ASyS from those involved in the later development of PSE. Early seizures are thought to result mainly from acute disruption of brain integrity and metabolic homeostasis, including excitatory glutamate release, electrolyte and acid-base disturbances, spreading depolarizations, oxidative stress, and secondary neuronal injury. These processes may create transient electrically irritable tissue in the acute phase of stroke [17,25,26].

**Figure 1:**
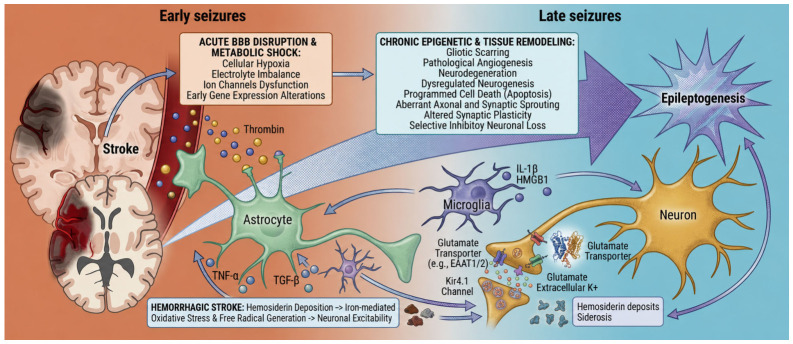
Mechanisms of post-stroke epileptogenesis, distinguishing mechanisms involved in early and late seizures. Legend: BBB: blood–brain barrier.

By contrast, late seizures and PSE reflect more durable epileptogenic changes. Blood–brain barrier (BBB) dysfunction appears to play a central role in this process. Extravasation of serum albumin into the brain parenchyma may activate TGF-β signaling in astrocytes, triggering inflammatory gene expression, perineuronal net degradation around inhibitory interneurons, and extracellular matrix remodeling. Together, these changes may increase neuronal excitability and reduce seizure threshold [26,27,28]. Persistent BBB dysfunction may also establish a self-reinforcing cycle, in which seizures further disrupt the BBB and perpetuate epileptogenic remodeling [26]. Other mechanisms, including chronic inflammation, gliotic scarring, angiogenesis, neurodegeneration, neurogenesis, axonal and synaptic sprouting, selective neuronal loss, and altered synaptic plasticity, may contribute to long-term seizure susceptibility [29,30]. In hemorrhagic stroke, hemosiderin deposition may additionally promote neuronal excitability through iron-mediated oxidative stress and free radical generation [25,26]. Figure 1 summarizes the mechanisms involved in post-stroke epileptogenesis.

Understanding these mechanisms is relevant because prevention of PSE would require early identification of epileptogenic changes and therapeutic interventions capable of inhibiting or reversing them [2]. This has motivated the investigation of biomarkers and potential therapeutic targets. However, methodological heterogeneity, variable definitions, and insufficient validation currently limit the translation of most biomarkers into routine clinical practice [31].

Among currently available tools, EEG is probably the most clinically accessible biomarker for PSE risk stratification. Epileptiform activity within the first seven days after stroke has been associated with a higher 5-year risk of epilepsy, and regional slowing also appears to increase risk. This is particularly relevant because many stroke survivors who later develop epilepsy do not have recognized ASyS [32]. Therefore, EEG may help identify a subgroup of patients who would otherwise be considered clinically silent from an epileptological perspective. Its role, however, should be interpreted as risk stratification rather than as an isolated indication for prophylactic ASM treatment.

Beyond EEG, blood and cerebrospinal fluid biomarkers have been investigated mainly in exploratory or translational settings. Different studies have associated PSE with markers of astrocytic activation, cellular stress, angiogenesis, inflammation, neuronal injury, inhibitory neurotransmission, and microRNA expression. Examples include downregulated S100B and Hsc70 combined with upregulated endostatin; increased TNFSF-14; elevated IL-6 and IL-1β; reduced cerebrospinal fluid GABA; and increased serum NSE and miR-155 [31,33,34,35]. Recent clinical and translational studies illustrate both the potential and the limitations of biomarker-guided prediction and preventive strategies.

Some combinations of biomarkers have shown better predictive performance than isolated measures, including IL-1β combined with the SeLECT score and combined detection of low GABA with elevated NSE and miR-155 [25,35]. In a prospective observational study of 915 patients with acute ischemic stroke, 53 developed PSE within one year; the combination of the SeLECT score and serum IL-1β improved predictive performance compared with either measure alone, supporting the concept that inflammatory biomarkers may complement clinical-imaging scores [36]. However, this finding requires external validation before clinical implementation.

More experimental approaches, including single-cell transcriptomic and bioinformatic analyses, have also identified candidate molecular pathways potentially involved in post-ischemic stroke epilepsy, such as Hsp90aa1, JUN, and Ccl2-related regulation of neural stem/progenitor cell migration and differentiation [37]. These data may help identify future therapeutic targets, but they still require clinical validation.

Taken together, current evidence supports a biologically plausible transition from acute stroke-related hyperexcitability to chronic epileptogenic remodeling in selected patients. For clinical practice, however, most molecular biomarkers should still be viewed as research tools. At present, EEG findings combined with clinical and imaging variables remain the most actionable strategy for PSE risk stratification.

## 5. Risk Factors

Risk stratification after stroke is clinically relevant because a seizure after a cerebrovascular insult may have different implications depending on timing, stroke subtype, lesion characteristics, and patient profile. Several studies have evaluated predictors of ASyS and PSE, showing that risk is not evenly distributed across stroke survivors. Table 1 summarizes selected studies grouped by stroke subtype. Studies that specifically generated or validated predictive scores are discussed in Section 6.

Table 1 shows that the risk of ASyS and PSE is not uniform across stroke survivors. ASyS are mainly associated with markers of acute injury and cortical irritability, such as hemorrhagic stroke, stroke severity, cortical involvement, and hemorrhagic transformation. PSE is more consistently associated with factors suggesting long-term epileptogenic potential, particularly cortical involvement, stroke severity, and previous ASyS, especially status epilepticus. CVT has a distinct risk profile, with acute seizures and intracranial bleeding complications playing a central role. These factors are clinically useful to define surveillance, EEG indication, discharge counseling, and follow-up strategy, but they should not be interpreted as automatic indications for prophylactic ASM treatment.

## 6. Predictive Scores

After the identification of clinical and imaging risk factors, several predictive scores were developed to estimate the risk of PSE in individual patients. Their main clinical value is not to indicate routine ASM prophylaxis, but to identify patients who may benefit from closer surveillance, a lower threshold for EEG, structured counseling, and more careful follow-up. These tools may also help stratify high-risk patients for future clinical trials evaluating preventive or disease-modifying strategies [10,45].

For ischemic stroke, the SeLECT score was the first widely used model to predict late seizures. It includes five routinely available variables: stroke severity, large-artery atherosclerotic etiology, early seizures, cortical involvement, and middle cerebral artery territory involvement [46]. Subsequent models refined this approach by incorporating more specific information about ASyS and EEG findings. SeLECT 2.0 distinguished short ASyS from acute symptomatic status epilepticus, recognizing the particularly high epileptogenic relevance of status epilepticus after ischemic stroke [1]. More recently, SeLECT-ASyS further incorporated the timing and type of ASyS, whereas SeLECT-EEG added early electrographic biomarkers, including epileptiform activity and regional slowing, to improve prediction in patients without clinically recognized ASyS [32,47].

From a practical perspective, these models should be selected according to the available clinical scenario. In patients with ischemic stroke without ASyS and without early EEG, SeLECT 2.0 may be applied. When ASyS occurs, SeLECT-ASyS is more appropriate because it accounts for seizure timing and type. When early EEG is available in patients without clinical ASyS, SeLECT-EEG may provide additional risk stratification. These scores are available in predictive models in practical web and smartphone applications to facilitate their use in clinical practice and are summarized in Table 2.

For hemorrhagic stroke, the CAVE score remains the most established tool for estimating the risk of PSE after ICH. It assigns one point each for cortical involvement, age < 65 years, hematoma volume > 10 mL, and early seizure within seven days of ICH [48]. Subsequent models have attempted to refine prediction by giving greater weight to cortical involvement, incorporating radiomics features, or proposing alternative clinical variables. These include CAVE2, radiomics-clinical models, and the LEAN score, which includes neurosurgical procedure, age < 65 years, lobar hemorrhage, and early seizures [49,50,51]. Although these newer models are promising, CAVE remains simple, clinically accessible, and widely recognized. It is demonstrated below in Table 3.

Prediction after CVT requires a distinct approach because its risk profile differs from that of arterial ischemic stroke and ICH. The DIAS^3^ score was developed to estimate epilepsy risk after CVT and includes clinical variables such as decompressive hemicraniectomy, intracerebral hemorrhage, age, acute seizures, acute status epilepticus, and subdural hematoma at presentation [52].

Overall, predictive scores should be interpreted as tools for risk stratification rather than as treatment algorithms. Even in patients with high predicted risk, current evidence does not support routine prophylactic ASM use solely based on a score. Their practical role is to identify patients who may require closer neurological monitoring, avoidance of neurotoxic medications in the acute phase, a lower threshold for EEG, more detailed discharge counseling, and structured follow-up. In this sense, predictive scores help translate epidemiological risk into clinical vigilance, without replacing individualized decision-making.

## 7. Acute Symptomatic Seizures

**Figure 2:**
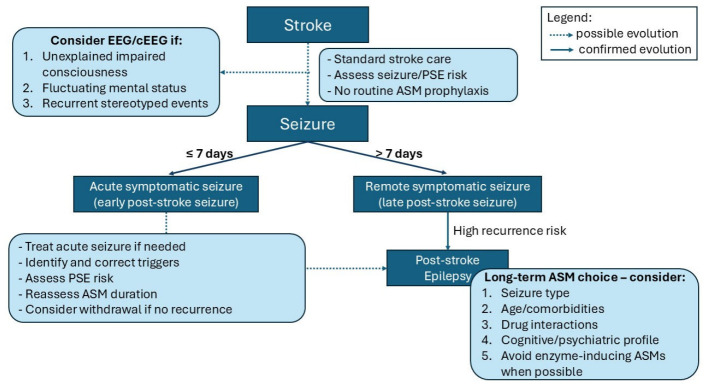
Practical framework for management of post-stroke seizures (acute symptomatic seizures and post-stroke epilepsy). Legend: ASM: antiseizure medication; EEG: electroencephalography; cEEG: continuous electroencephalography; PSE: post-stroke epilepsy. Dashed arrows indicate possible evolution or risk-based pathways; solid arrows indicate diagnostic or management pathways.

The first management question after stroke is whether ASM should be used to prevent seizures before any clinical or electrographic event has occurred. Current evidence does not support routine primary ASM prophylaxis in unselected stroke patients, and available data suggest that ASM treatment does not prevent the later development of PSE [6]. However, this should be distinguished from the treatment of documented ASyS, which are associated with a higher risk of epilepsy and worse outcomes [17]. Figure 2 summarizes a practical framework for managing seizures across the continuum from acute symptomatic seizures to established PSE.

Several pharmacological strategies have been investigated for seizure prevention or antiepileptogenesis after stroke. These include antiseizure-based approaches, such as levetiracetam or eslicarbazepine, and non-ASM strategies with potential antiepileptogenic or vascular effects, such as angiotensin receptor blockers and statins [8,53,54,55,56,57]. Although these studies are relevant for future preventive strategies, they do not currently change the general recommendation against routine primary prophylaxis.

Beyond specific drugs, optimized stroke care may influence the risk of later epilepsy by reducing infarct size, hemorrhagic complications, and secondary brain injury. Revascularization therapies in ischemic stroke have been associated with a lower risk of epilepsy in observational data, and implementation of organized stroke systems of care may also reduce PSE risk [11,58]. Pediatric data are more limited and less consistent, reinforcing the need to interpret preventive strategies according to age group, stroke mechanism, and clinical context [59].

Guidelines are broadly consistent in discouraging routine ASM prophylaxis for most stroke patients without seizures, although recommendations vary according to stroke subtype and specific high-risk scenarios. In patients with spontaneous ICH, ASMs are recommended when clinical or electrographic seizures are confirmed, and continuous EEG is reasonable in patients with unexplained or fluctuating mental status. In aneurysmal subarachnoid hemorrhage, short-term prophylaxis may be considered in selected patients with high-risk features, whereas prophylaxis is not beneficial in low-risk patients. In cerebral venous thrombosis, ASM treatment is suggested for patients with supratentorial lesions and acute seizures to prevent early recurrence [21,60,61,62,63,64]. These recommendations are summarized in Table 4.

Once an ASyS or electrographic seizure occurs, ASM treatment is usually indicated. At this point, the main uncertainty is not whether the event should be treated acutely, but how long treatment should be continued. This decision should be guided by the underlying etiology, particularly its reversibility, the risk of subsequent epilepsy, and the expected duration of post-injury hyperexcitability. The latter is particularly relevant for determining treatment duration after ASyS [65].

For ASyS due to structural etiologies, evidence is insufficient to define the optimal duration of ASM therapy. Guideline-endorsed strategies often favor early withdrawal after 7–14 days, whereas real-world practice frequently extends treatment for 3–12 months [66]. In stroke patients, because hyperexcitability may persist for weeks to months and the underlying lesion is often permanent, a pragmatic approach may be to maintain ASM treatment for approximately 3–6 months followed by individualized reassessment, particularly when seizures were severe, recurrent, electrographic, or associated with high-risk structural features [65].

ASM choice should follow general principles, including seizure type, age, comorbidities, renal and hepatic function, potential drug interactions, route of administration, and local availability. In hospitalized stroke patients, drugs that can be rapidly titrated, administered intravenously, and used with minimal drug–drug interactions are often preferred. Levetiracetam is commonly used in this context because it fulfills many of these practical requirements, although this preference reflects clinical feasibility more than proven superiority for the prevention of PSE.

Therefore, the lack of evidence supporting routine prophylaxis should not preclude appropriate treatment after documented clinical or electrographic seizures.

## 8. Post-Stroke Epilepsy Management

**Figure 3:**
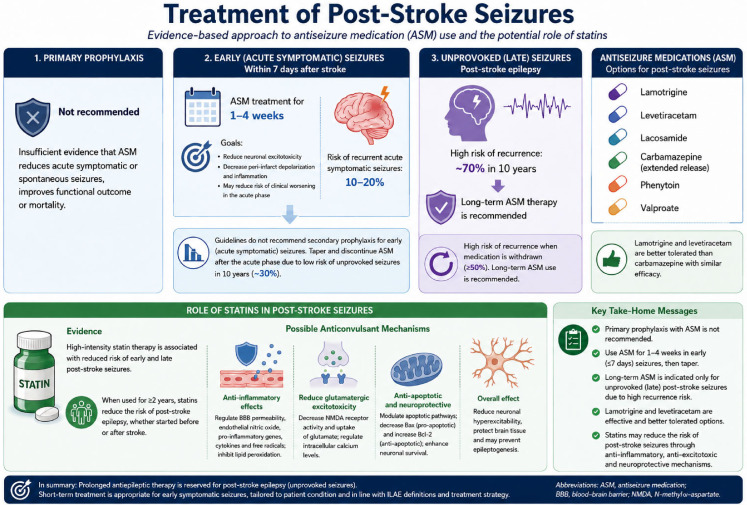
Treatment of post-stroke seizures: role of primary prophylaxis, differences in management of early and late seizures, antiseizure medication options, possible role of statins in post-stroke seizures, and key take-home messages.

Patients with PSE often require long-term ASM treatment. Therefore, drug choice should consider not only seizure control but also tolerability, comorbidities, cognitive and psychiatric vulnerability, drug interactions, and the vascular profile of the patient. Adverse effects such as fatigue, dizziness, behavioral symptoms, and depression may be particularly relevant in stroke survivors, who often already have functional limitations and polypharmacy [10]. Enzyme-inducing ASMs, such as phenobarbital, phenytoin, and carbamazepine, deserve special caution because they may reduce the efficacy of concomitant medications and have been associated with increased mortality [67]. This is particularly important when interactions involve secondary stroke prevention, including oral anticoagulants. Figure 3 summarizes the management of post-stroke seizures, highlighting the role of primary prophylaxis, the differences in management of early and late seizures, antiseizure medication options, as well as the possible role of statins in post-stroke seizures and key take-home messages.

Available evidence does not identify a single preferred ASM for all patients with PSE, but it supports some practical principles. In observational data from Sweden, levetiracetam was the most frequently used ASM for treatment initiation and showed the highest persistence in adults with PSE [68]. In the broader context of focal epilepsy, SANAD II supported lamotrigine as a first-line option, and a network meta-analysis suggested that levetiracetam and lamotrigine have favorable safety and tolerability profiles [69,70]. In cohort data specifically evaluating PSE, lamotrigine was associated with lower mortality than carbamazepine, whereas valproic acid was associated with higher cardiovascular and all-cause mortality; levetiracetam was associated with lower cardiovascular mortality than carbamazepine, without a significant difference in overall mortality [71]. Other newer-generation ASMs, including lacosamide and eslicarbazepine, may also be well tolerated and useful in selected patients [72].

In practical terms, levetiracetam is attractive in the acute or early post-stroke setting because it can be rapidly titrated, has few drug interactions, and is available intravenously. Lamotrigine may be particularly suitable for chronic focal epilepsy when slow titration is feasible and tolerability is a priority. Newer-generation ASMs are often preferred over older enzyme-inducing drugs in patients receiving secondary vascular prevention, anticoagulation, or multiple medications.

In older adults, ASM selection requires particular caution because tolerability may be reduced by age-related changes in pharmacokinetics and pharmacodynamics, comorbidities, renal or hepatic impairment, and polypharmacy [68]. Enzyme-inducing ASMs deserve special attention in this population because of their potential interactions with antithrombotic agents, statins, antihypertensive drugs, and other medications commonly used for vascular prevention. Cognitive and neuropsychiatric adverse effects should also be considered, particularly with drugs such as topiramate, phenobarbital, or benzodiazepines, which may worsen cognition, gait stability, or sedation in vulnerable patients. Overall, treatment in older patients should be individualized, usually following a “start low and go slow” approach. In this subgroup, lamotrigine, levetiracetam, and lacosamide meet many of the preferred criteria for ASM selection, including favorable tolerability, low interaction potential, and suitability for focal epilepsy, although renal function, cardiac comorbidities, and concomitant medications should guide the final choice.

On the other hand, pediatric PSE treatment should not be managed by direct extrapolation from adult data. In one pediatric cohort, carbamazepine was associated with better seizure control and higher ASM discontinuation rates than levetiracetam [59].

Most patients with PSE can be managed pharmacologically, but drug resistance may occur. The risk of drug resistance appears to be highest when PSE develops within the first months after stroke and decreases progressively thereafter, particularly after the first year [73]. In selected patients with drug-resistant PSE, epilepsy surgery or other advanced therapeutic strategies may be considered. Careful presurgical evaluation is essential, and appropriate surgical selection can lead to favorable outcomes in selected cases [74].

Once seizure freedom is achieved, ASM withdrawal becomes another relevant clinical question. Decisions should consider the duration of seizure freedom, seizure severity, adverse effects, neurological examination, EEG findings, and patient preference [60]. However, withdrawal in PSE is particularly complex because this is a lesional epilepsy with a substantial risk of recurrence after discontinuation. Features arguing against withdrawal include focal seizures, short seizure-free interval, abnormal neurological examination, and epileptiform EEG activity [75]. Therefore, ASM withdrawal should be individualized and approached cautiously rather than routinely pursued.

## 9. Future Directions

As stroke care improves and the number of stroke survivors increases, greater attention should be directed to long-term complications, including PSE. Future research should move beyond risk description toward the identification of patients who may benefit from early interventions capable of modifying epileptogenesis. Artificial intelligence and machine learning models may help in this process by integrating clinical variables, stroke severity, lesion location and volume, EEG features, neuroimaging markers of BBB dysfunction, and circulating biomarkers. Such multimodal approaches may be particularly useful for identifying patients at sufficiently high risk to justify intensive EEG monitoring, structured follow-up, or inclusion in preventive trials. However, these models will require external validation, transparent reporting, calibration across different stroke populations, and feasibility testing in real-world settings before they can be incorporated into routine practice.

Anti-inflammatory and disease-modifying strategies also represent an important research frontier. The IL-1β pathway is biologically plausible because it contributes to neuroinflammation, BBB dysfunction, and neuronal hyperexcitability. Similarly, the TGF-β pathway is relevant after vascular injury because albumin extravasation through a disrupted BBB may activate astrocytic TGF-β signaling and downstream epileptogenic remodeling. Nevertheless, clinical translation remains limited. IL-1β-targeted therapies have been explored mainly in pharmacoresistant inflammatory epilepsies and status epilepticus, whereas robust trials demonstrating prevention of PSE are lacking. TGF-β modulation, including indirect approaches such as angiotensin receptor blockade, remains supported mainly by experimental or observational data rather than definitive clinical evidence. Therefore, these pathways should currently be regarded as promising mechanistic targets rather than established preventive therapies.

Several concrete gaps should be prioritized. First, future studies should harmonize definitions of ASyS, remote symptomatic seizures, and PSE, as variable definitions still limit comparability across cohorts. Second, prediction models should be validated across ischemic stroke, ICH, subarachnoid hemorrhage, CVT, pediatric stroke, and older populations. Third, biomarkers should be evaluated in prospective, adequately powered cohorts with standardized timing of collection and clinically meaningful endpoints. Fourth, preventive trials should enrich recruitment for patients at high epileptogenic risk, such as those with cortical involvement, severe stroke, hemorrhagic transformation or ICH, ASyS/status epilepticus, epileptiform EEG abnormalities, or markers of persistent BBB dysfunction. Finally, future trials should distinguish short-term seizure suppression from true antiepileptogenesis and should include outcomes relevant to patients, including cognition, functional recovery, quality of life, drug tolerability, and vascular safety.

International collaboration, such as the International Post Stroke Epilepsy Research Consortium (IPSERC) [2], may accelerate progress by harmonizing definitions, validating biomarkers, improving risk stratification, and supporting preventive trials. However, PSE is heterogeneous, and management is influenced not only by stroke subtype and patient profile but also by local access to EEG, advanced neuroimaging, and biomarkers. For this reason, simple clinical frameworks remain essential to translating emerging evidence into real-world practice.

## 10. Conclusions

Distinguishing ASyS from PSE is essential for prognosis, counseling, EEG use, ASM treatment, treatment duration, and follow-up. Although current evidence does not support routine primary ASM prophylaxis after stroke, documented clinical or electrographic seizures should be appropriately treated, and established PSE often requires long-term individualized therapy. Risk stratification, clinical context, and patient-specific factors should guide monitoring and treatment decisions.

## Figures and Tables

**Table 1:** Selected studies evaluating risk factors for acute symptomatic seizures (ASyS) and post-stroke epilepsy (PSE) according to stroke subtype.

Stroke Subtype	Study	Population Specification	Sample Size	Evaluated Outcome	Identified Risk Factors	Clinical Interpretation/Notes
Ischemic stroke	Ferreira-Atuesta et al., 2021 [38]	Adults	4229	ASyS and PSE	AsyS: stroke severity, posterior cerebral artery territory infarct, and large-artery atherosclerosis etiology. PSE: AsyS, stroke severity, cortical involvement, and large-artery atherosclerosis etiology.	Early EEG was not predictive of PSE. Reperfusion treatment was not associated with the risk of AsyS or PSE. Small-vessel occlusion strokes carried a low risk of AsyS.
	Alet et al., 2022 [39]	Adults	1586	PSE	ASyS, cortical involvement, Fazekas scale score, and mRS at discharge	-
	Pozo Putalivo et al., 2025 * [40]	Adults	693	PSE	Temporal lobe involvement, NIHSS ≥ 11, MCA territory, early seizures, cortical involvement, presence of large-artery atherosclerosis, and SeLECT score ≥ 4.	-
	Verburgt et al., 2025 * [10]	Young adults (<50 years)	1231	PSE	ASyS and cortical involvement	-
Hemorrhagic stroke	Lahti et al., 2017 [41]	Patients with primary ICH surviving > 3 months	615	PSE	Subcortical hematoma location and early seizures.	The risk of new-onset PSE was highest during the first year.
	Zöllner et al., 2020 [42]	Patients with ICH or SAH	19,331	ASyS	Presence of an acute non-neurological infection and a lower premorbid functional level.	ASyS are equally common in ICH and SAH patients in this registry.
	Pezzini et al., 2024 [30]	Adults with spontaneous ICH	2570	ASyS and PSE	ASyS: lobar location of the hematoma. PSE: occurrence of ASyS.	ASyS were divided into short seizures and SE. Hematoma volume was related to short seizures. Lobar location, increasing admission NIHSS score, and surgical evacuation of hematoma were associated with SE.
	Pozo Putalivo et al., 2025 * [40]	Adults	67	PSE	Bleeding > 10 mL, early seizures, cortical involvement, and CAVE score ≥ 3.	Higher cumulative risk was observed in hemorrhagic stroke, particularly with higher CAVE scores.
	Verburgt et al., 2025 * [10]	Young adults (<50 years) after ICH	157	PSE	Cortical involvement	-
Cerebral venous thrombosis (CVT)	Lindgren et al., 2020 [43]	Adults	1281	ASyS	ICH, cerebral edema/infarction without ICH, cortical vein thrombosis, superior sagittal sinus thrombosis, focal neurologic deficit, sulcal SAH, female-specific risk factors	No subgroup was identified with sufficient risk of ASyS to justify prophylactic ASM treatment.
	Sánchez van Kammen et al., 2020 [44]	Adults	1127	PSE	SE in acute phase, decompressive hemicraniectomy, acute seizures, subdural hematoma, and intracerebral hemorrhage	During a median follow-up of 2 years, approximately 10% of patients with CVT developed PSE.

Legend: ASMs: antiseizure medications; ASyS: acute symptomatic seizures; CVT: cerebral venous thrombosis; EEG: electroencephalography; ICH: intracerebral hemorrhage; MCA: middle cerebral artery; mRS: modified Rankin Scale; NIHSS: National Institutes of Health Stroke Scale; PSE: post-stroke epilepsy; SAH: subarachnoid hemorrhage; SE: status epilepticus. * Studies including both ischemic and hemorrhagic stroke populations.

**Table 2:** Comparison of predictive scores (SeLECT, SeLECT 2.0, SeLECT-ASyS, and SeLECT-EEG) for post-stroke epilepsy after ischemic stroke.

SeLECT	Pt	SeLECT 2.0	Pt	SeLECT-ASyS	Pt	SeLECT-EEG	Pt
Cortical involvement	2	Cortical involvement	2	Cortical involvement	1	Cortical involvement	2
Large-artery atherosclerosis	1	Large-artery atherosclerosis	1	Large-artery atherosclerosis and female	1	Large-artery atherosclerosis	1
				Large-artery atherosclerosis and male	2		
NIHSS score 4–10	1	NIHSS score 4–10	1	FBTC, day 1 to 7	1	NIHSS score 4–10	1
NIHSS score ≥ 11	2	NIHSS score ≥ 11	2	Other types of short seizures, day 1 to 7	0	NIHSS score ≥ 11	2
Territory of MCA involvement	1	Territory of MCA involvement	1	FBTC, same day of stroke onset	2	Territory of MCA involvement	1
Any acute symptomatic seizure	3	Short acute symptomatic seizure	3	Other type of short seizure, same day of stroke onset	1	Regional slowing	1
		Acute symptomatic status epilepticus	7	Acute symptomatic status epilepticus	4	All epileptiform activities (IED, GPD, LPD, LRDA, ESei, E-SE)	1
**Maximum**	**9**	**Maximum**	**13**	**Maximum**	**7**	**Maximum**	**8**
**Practical use:** Baseline PSE risk after ischemic stroke		**Practical use:** Refines risk by distinguishing short seizure vs. status epilepticus		**Practical use:** Preferred when ASyS occurred		**Practical use:** Adds risk information in clinically silent patients	

Legend: ASyS = acute symptomatic seizure; E-SE = electrographic status epilepticus; ESei = electrographic seizures; FBTC: focal to bilateral tonic–clonic seizure; GPD = generalized periodic discharges; IED = interictal epileptic activity; LPD = lateralized periodic discharges; LRDA = lateralized rhythmic delta activity; MCA = middle cerebral artery; NIHSS = National Institutes of Health Stroke Scale; PSE: post-stroke epilepsy.

**Table 3:** CAVE score for prediction of post-stroke epilepsy after intracerebral hemorrhage.

CAVE	Pt
Cortical involvement of ICH	1
Age < 65 years	1
Volume of ICH > 10 mL	1
Early seizure within 7 days of ICH	1
**Maximum**	**4**
**Interpretation:** 0 points: 0.6% risk of PSE; 1 point: 3.6% risk of PSE; 2 points: 9.8% risk of PSE; 3 points: 34.8% risk of PSE; 4 points: 46.2% risk of PSE	

Legend: ICH: intracerebral hemorrhage; PSE: post-stroke epilepsy.

**Table 4:** Summary of guideline recommendations for acute symptomatic seizure management after stroke.

Stroke Subtype	Guideline Author (Publication Year)	Recommendations (Class/Strength of Recommendation)
Ischemic stroke	AHA/ASA (2026) [60]	- Prophylactic treatment with ASM is not recommended (Class 3).
Intracerebral hemorrhage (ICH)	AHA/ASA (2022) [61]	- In patients with impaired consciousness and confirmed electrographic seizures, ASM should be administered (Class 1). - In patients with clinical seizures, ASM treatment is recommended (Class 1). - In patients with unexplained abnormal or fluctuating mental status or suspicion of seizures, continuous EEG (>24 h) is reasonable (Class 2a). - In patients without evidence of seizures, prophylactic ASM is not beneficial (Class 3).
	ESO (2025) [64]	- It suggests against treatment with ASMs for the primary prevention of acute/remote symptomatic epileptic seizures (Weak against intervention). - In ASyS, it cannot make a recommendation about the use of ASM. - In adults in whom ASM was initiated after ASyS, and in whom no further seizures occur, it suggests that ASM treatment be discontinued from 4 weeks onwards (Expert consensus statement).
Subarachnoid hemorrhage (SAH)	AHA/ASA (2023) [62]	- In patients with fluctuating neurological examination, depressed mental state, ruptured MCA aneurysm, high-grade SAH, ICH, hydrocephalus, or cortical infarction, cEEG monitoring is reasonable (Class 2a). - In patients with high-seizure-risk features (i.e., ruptured MCA aneurysm, high-grade SAH, ICH, hydrocephalus, and cortical infarction), use of prophylactic ASM may be reasonable (Class 2b). - In patients without high-seizure-risk features, prophylactic treatment with ASM is not beneficial (Class 3). - Phenytoin for seizure prevention and/or antiseizure prophylaxis is associated with excess morbidity and mortality (Class 3). - In patients who present with seizures, treatment with ASM for ≤7 days is reasonable (Class 2a). - In patients without prior epilepsy who present with seizures, treatment with ASM over 7 days is not effective for reducing future SAH-associated seizure risk (Class 3).
Cerebral venous thrombosis (CVT)	ESO (2017) [63]	- Suggest using ASMs in patients with acute CVT with supratentorial lesions and seizures to prevent early recurrent seizures (Weak).

Legend: AHA/ASA: American Heart Association/American Stroke Association; aSAH: aneurysmal subarachnoid hemorrhage; ASM: antiseizure medication; ASyS: acute symptomatic seizure; cEEG: continuous electroencephalography; EEG: electroencephalography; ESO: European Stroke Organization; MCA: middle cerebral artery.

## Data Availability

No new data were created or analyzed in this study. Data sharing is not applicable to this article.

## Abbreviations

ASM	antiseizure medication
ASyS	acute symptomatic seizures
BBB	blood–brain barrier
CVT	cerebral venous thrombosis
EEG	electroencephalography
ICH	intracerebral hemorrhage
PSE	post-stroke epilepsy
